# A Systematic Review and Meta-Analysis of Serum Concentrations of Ischaemia-Modified Albumin in Acute Ischaemic Stroke, Intracerebral Haemorrhage, and Subarachnoid Haemorrhage

**DOI:** 10.3390/biom12050653

**Published:** 2022-04-29

**Authors:** Arduino A. Mangoni, Angelo Zinellu

**Affiliations:** 1 Discipline of Clinical Pharmacology, College of Medicine and Public Health, Flinders University, Sturt Road, Bedford Park, SA 5042, Australia; 2 Department of Clinical Pharmacology, Flinders Medical Centre, Southern Adelaide Local Health Network, Flinders Drive, Bedford Park, SA 5042, Australia; 3 Department of Biomedical Sciences, University of Sassari, 07100 Sassari, Italy; azinellu@uniss.it

**Keywords:** ischaemia-modified albumin, stroke, acute ischaemic stroke, intracerebral haemorrhage, subarachnoid haemorrhage, biomarkers

## Abstract

The identification of robust circulating biomarkers of stroke may improve outcomes. We conducted a systematic review and meta-analysis of serum concentrations of ischaemia-modified albumin (IMA) in subjects with or without acute ischaemic stroke (AIS), intracerebral haemorrhage (ICH), and subarachnoid haemorrhage (SAH). We searched PubMed, Web of Science, Scopus, and Google Scholar from inception to March 2022. Risk of bias and certainty of evidence were assessed using the Joanna Briggs Institute Critical Appraisal Checklist and GRADE, respectively. In 17 studies, IMA concentrations were significantly higher in patients with AIS (standard mean difference, SMD = 2.52, 95% CI 1.92 to 3.12; *p* < 0.001), ICH (SMD = 3.13, 95% CI 1.00 to 5.25; *p* = 0.004), and SAH (SMD = 4.50, 95% CI 0.91 to 7.01; *p* = 0.014) vs. controls (very low certainty of evidence). In AIS, the effect size was associated with the male gender, and was relatively larger in studies conducted in Egypt and India and those using enzyme-linked immunosorbent assays. IMA concentrations were progressively higher, by direct comparison, in SAH, ICH, and AIS. In sensitivity analysis, the pooled SMDs were not altered when individual studies were sequentially removed. Our meta-analysis suggests that IMA concentrations might be useful to diagnose stroke and discriminate between AIS, ICH, and SAH (PROSPERO registration number: CRD42021320535).

## 1. Introduction

Stroke represents the acute neurological injury that results from brain ischaemia, particularly acute ischaemic stroke (AIS), and brain haemorrhage secondary to intracerebral haemorrhage (ICH) or subarachnoid haemorrhage (SAH). AIS, ICH, and SAH account for 62%, 28%, and 10% of strokes worldwide, respectively [1,2]. Despite significant advances in diagnosis and management, stroke remains the second leading cause of mortality and disability globally, and accounts for 3–7% of the total healthcare costs in developed countries with an estimated cost per person of USD 140,048 [3,4]. A critical determinant of clinical outcomes is the early diagnosis of the specific stroke subtype, which allows the prompt institution of reperfusion therapies (AIS), treatment of specific haemodynamic parameters (AIS, ICH, and SAH), or surgical intervention (AIS and SAH) [5,6,7,8]. A correct diagnosis, primarily using brain imaging, is also important as different stroke sub-types share common risk factors, e.g., hypertension and diabetes, and their clinical presentation may mimic other neurological disorders such as migraine, epilepsy, and brain tumours [5,6,7,8]. However, rapid access to imaging facilities can be challenging in remote areas, low-income countries, or when the clinical presentation is particularly subtle. This has prompted the search for additional biomarkers for the diagnosis of stroke. Ideally, such biomarkers should be easily measurable and interpretable, accurate, reproducible, and, if possible, guide appropriate management strategies [9]. 

Several blood proteins have been investigated for their potential use as stroke biomarkers in view of their ability to reflect specific pathophysiological processes occurring in the brain, e.g., glial activation, neuronal injury, altered haemostasis, endothelial dysfunction, inflammation, and apoptosis [10,11,12]. Albumin, one of the most abundant circulating proteins, undergoes chemical modifications of the N-terminal sequence during ischaemic conditions, which lead to the generation of ischaemia-modified albumin (IMA). Such modifications are thought to be the result of a state of oxidative stress, increased production of reactive oxygen species, and acidosis, typically associated with ischaemia [13]. Compared to parent albumin, IMA exhibits a reduced binding capacity for metals, particularly copper, nickel, and cobalt [13]. Serum concentrations of IMA increase within 24 h in AIS and then gradually decrease over the following week [14,15]. Similar increases, albeit of a smaller magnitude, have also been observed in ICH and SAH [16], suggesting the potential role of IMA as a biomarker of stroke as well as a tool to differentiate between AIS, ICH, and SAH. We sought to investigate this issue by conducting a systematic review and meta-analysis of serum IMA concentrations in subjects with or without AIS, ICH, and SAH. The primary hypothesis was to demonstrate the presence of significant differences in IMA concentrations between patients with AIS, ICH, or SAH and non-stroke controls. In addition, we sought to determine the presence of differences in IMA concentrations between AIS, ICH, and SAH patients.

## 2. Materials and Methods

### 2.1. Literature Search and Study Selection

A systematic literature search was conducted in PubMed, Web of Science, Scopus and Google Scholar, from inception to March 2022, using the following terms: “acute ischaemic stroke” or “intracerebral haemorrhage” or “brain haemorrhage” or “subarachnoid haemorrhage” and “ischaemia-modified albumin”. Abstracts were independently screened by two investigators. If relevant, the full text was reviewed. Eligibility criteria were: (i) assessment of ischaemia-modified albumin in serum; (ii) comparison of subjects with or without AIS, ICH, and SAH (case-control design); (iii) sample size ≥10 participants; (iv) English language, and (v) full-text available. The references of the retrieved articles were also searched for additional studies. Any disagreement between the reviewers was resolved by a third investigator. The risk of bias was assessed using the Joanna Briggs Institute (JBI) Critical Appraisal Checklist for analytical studies (low, moderate, and high risk was indicated by a score of ≥5, 4, and <4, respectively) [17]. The certainty of evidence was assessed using the Grades of Recommendation, Assessment, Development and Evaluation (GRADE) Working Group system, which considers the study design, the risk of bias, the presence of unexplained heterogeneity, the indirectness of evidence, the imprecision of the results, the effect size [18], and the probability of publication bias [19]. The study complied with the Preferred Reporting Items for Systematic reviews and Meta-Analyses (PRISMA) 2020 statement (Supplementary Tables S1 and S2) [20]. The protocol was registered in the International Prospective Register of Systematic Reviews (PROSPERO, CRD42021320535).

### 2.2. Statistical Analysis

As different units of measurement (U/mL, absorbance units, pg/mL, or g/dL) were used to express IMA concentrations, standardised mean differences (SMDs) and 95% confidence intervals (CIs) were used to build forest plots of continuous data and to evaluate differences in serum IMA concentrations between participants with and without AIS, ICH, and SAH (significance level at *p* < 0.05). If necessary, means and standard deviations were extrapolated from medians and interquartile ranges [21]. Heterogeneity of SMD across studies was tested using the Q statistic (significance level at *p* < 0.10). An I^2^ value < 30% indicated no or slight heterogeneity whereas I^2^ ≥ 30% indicated moderate or substantial heterogeneity [22]. A random-effect model based on the inverse-variance method was used in the presence of moderate or substantial heterogeneity [22]. Sensitivity analysis was performed to investigate the influence of each study on the overall risk estimate [23]. Begg’s adjusted rank correlation test and Egger’s regression asymmetry test were used to assess publication bias (significance level at *p* < 0.05) [24,25]. The Duval and Tweedie “trim and fill” procedure was performed to further test and eventually correct the occurrence of publication bias. This method ‘trims’ (removes) smaller studies, causing funnel plot asymmetry, uses the trimmed funnel plot to estimate the true ‘centre’ of the funnel, then replaces the omitted studies and their missing ‘counterparts’ around the centre (filling). Besides providing an estimate of the number of missing studies, an adjusted intervention effect is derived by performing a meta-analysis including the filled studies [26]. Univariate meta-regression analyses were conducted to investigate associations between effect size and the following study and patient characteristics: age, the proportion of males, history of diabetes and hypertension, year of publication, sample size, and the country where the study was conducted. Statistical analyses were performed using Stata 14 (STATA Corp., College Station, TX, USA). 

## 3. Results

### 3.1. Systematic Research

**Figure 1:**
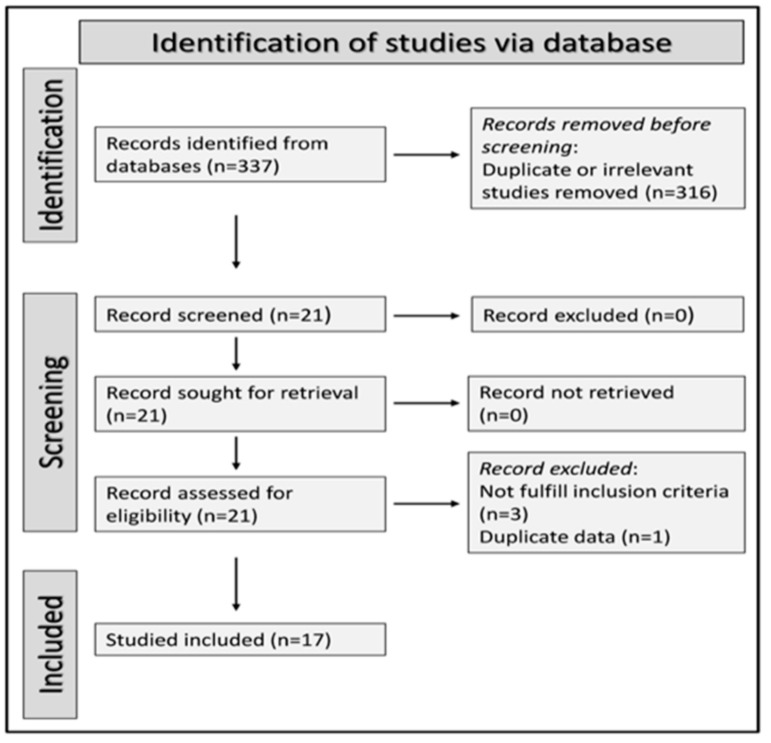
PRISMA 2020 flow diagram.

We identified 337 studies. A total of 316 were excluded after the initial screening because they were either duplicates or irrelevant. After a full-text review of the remaining 21 articles, four were further excluded because they did not fulfil the inclusion criteria or presented duplicate data. Thus, 17 studies were included in the final analysis (Figure 1 and Table 1) [15,16,27,28,29,30,31,32,33,34,35,36,37,38,39,40,41]. In all studies, the diagnosis of AIS, ICH, or SAH was made according to current professional recommendations and serum IMA was measured within 24 h of symptom onset. Most studies, 15 out of 17, were conducted in Turkey (*n* = 6), China (*n* = 3), India (*n* = 4), and Egypt (*n* = 2).

### 3.2. Acute Ischaemic Stroke

#### 3.2.1. Studies Selected

Seventeen studies reported IMA concentrations in 1136 AIS patients and 988 controls (Table 1). 

#### 3.2.2. Risk of Bias

The risk of bias was low in all studies (Table 2).

#### 3.2.3. Results of Individual Studies and Syntheses

**Figure 2:**
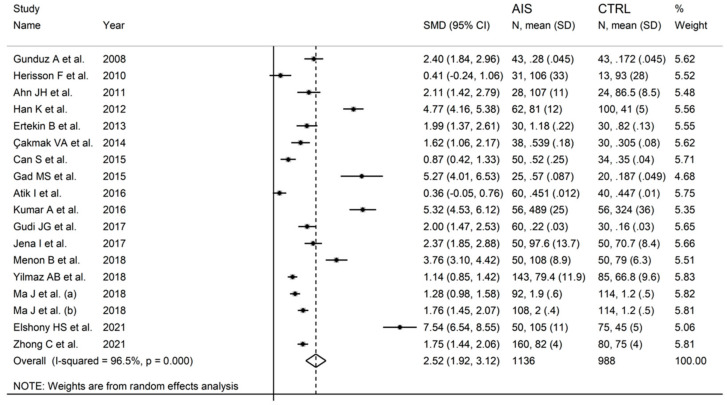
Forest plot of studies examining ischaemia-modified albumin in patients with acute ischaemic stroke and controls.

**Figure 3:**
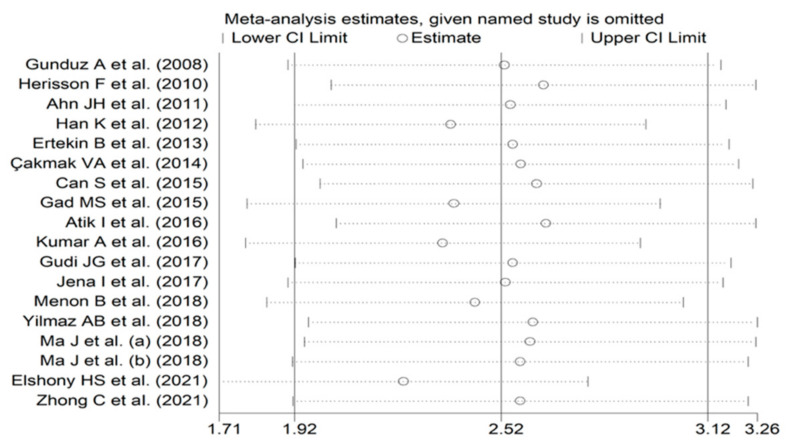
Sensitivity analysis of the association between ischaemia-modified albumin and acute ischaemic stroke. For each study, the effect size (hollow circles) corresponds to an overall effect derived from a meta-analysis excluding that study.

The forest plot of IMA concentrations in AIS patients and control subjects is shown in Figure 2. IMA concentrations were higher in AIS patients in all studies (mean difference range, 0.36 to 7.54) although the difference was not significant in two [28,35]. A random-effects model was used in view of the substantial heterogeneity observed (I^2^ = 96.5%, *p* < 0.001). Pooled results showed that IMA concentrations were significantly higher in AIS (SMD = 2.52, 95% CI 1.92 to 3.12; *p* < 0.001). In sensitivity analysis, the corresponding pooled SMD values were not substantially altered when each study was sequentially omitted (effect size range, between 2.35 and 2.65; Figure 3). 

#### 3.2.4. Publication Bias

**Figure 4:**
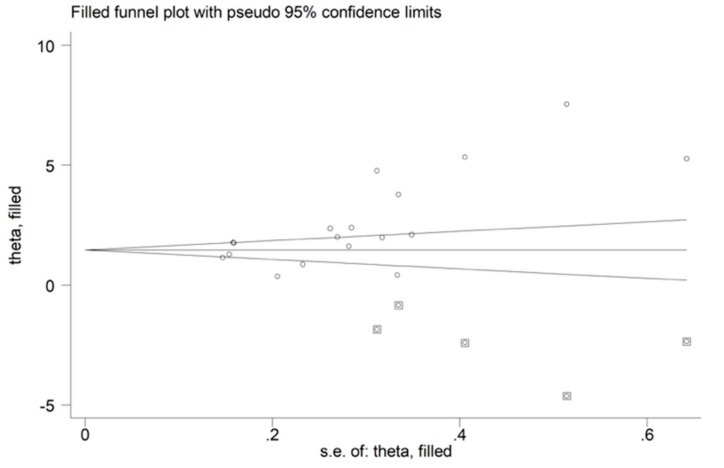
Funnel plot of ischaemia-modified albumin concentrations in patients with acute ischaemic stroke and controls after “trimming-and-filling”. Dummy studies and genuine studies are represented by enclosed circles and free circles, respectively.

There was a significant publication bias according to the Begg’s (*p* = 0.004) and Egger’s test (*p* = 0.002). Accordingly, the “trim-and-fill” method identified five potential missing studies to be added to the left of the funnel plot to ensure symmetry (Figure 4). This resulted in a reduced, albeit significant, effect size (SMD = 1.51, 95% CI 0.80–2.21; *p* < 0.001).

#### 3.2.5. Meta-Regression and Subgroup Analysis

**Figure 5:**
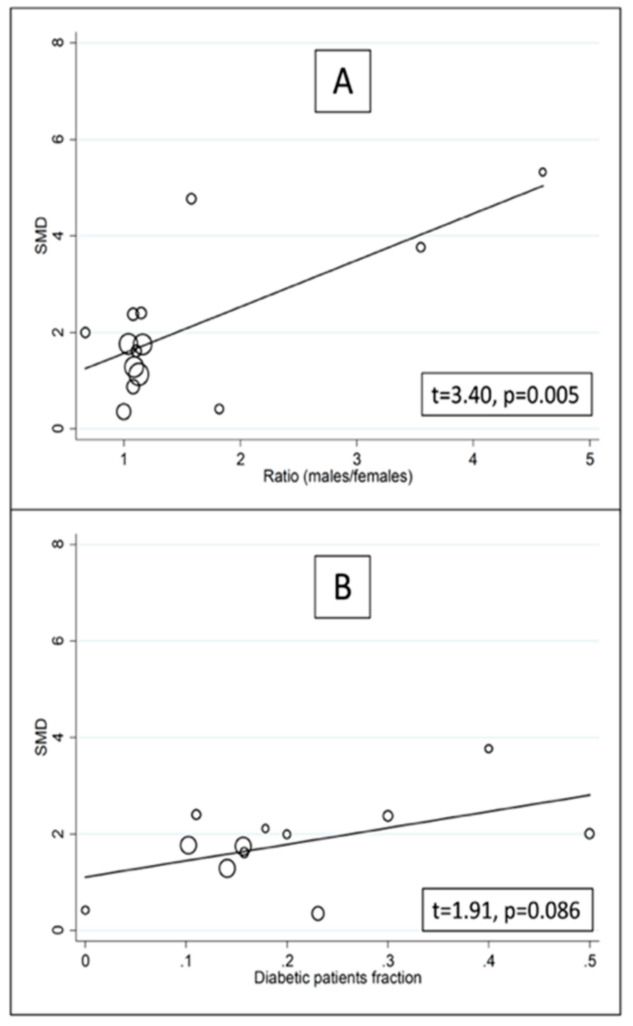
Univariate meta-regression analysis between effect size, proportion of males (**A**) and diabetes (**B**).

**Figure 6:**
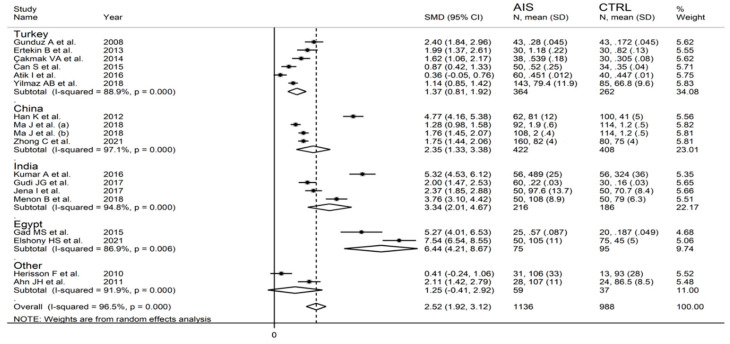
Forest plot of studies examining ischaemia-modified albumin in acute ischaemic stroke according to the country where the study was conducted.

**Figure 7:**
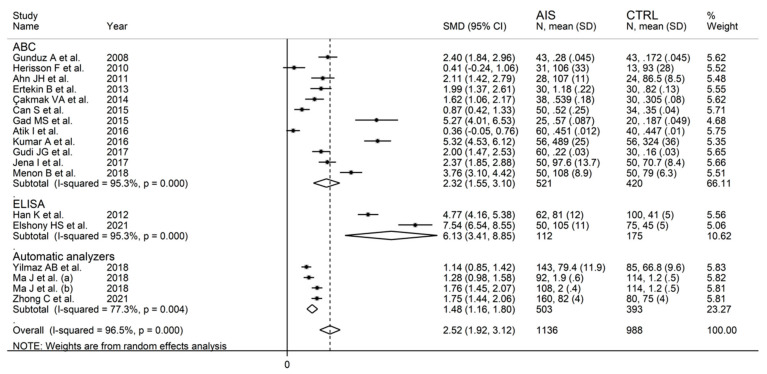
Forest plot of studies examining ischaemia-modified albumin in acute ischaemic stroke according to the assay method.

In univariate meta-regression, there were no significant associations between effect size and age (t = −0.67, *p* = 0.52), publication year (t = 0.85, *p* = 0.41), sample size (t = −0.97, *p* = 0.37), or hypertension (t = −0.40, *p* = 0.70). By contrast, there was a significant association with the proportion of males (t = 3.40, *p* = 0.005) and a non-significant trend with diabetes (t = 1.91, *p* = 0.086) (Figure 5). In sub-group analysis (Figure 6), the effect size was relatively larger in studies conducted in Egypt (SMD = 6.44, 95% CI 4.21 to 8.67) and India (SMD = 3.34, 95% CI 2.01 to 4.67; *p* < 0.001) when compared to China (SMD = 2.35, 95% CI 1.33 to 3.38; *p* < 0.001) or Turkey (SMD = 1.37, 95% CI 0.81 to 1.92; *p* < 0.001). Heterogeneity remained substantial, between 86.9% and 97.1%, in all sub-groups. Furthermore, the effect size was relatively larger in studies using enzyme-linked immunosorbent assays (SMD = 6.13, 95% CI 3.41 to 8.85) compared to albumin cobalt-binding spectrophotometric assays (SMD = 2.32, 95% CI 1.55 to 3.10) or automatic analysers (SMD = 1.48, 95% CI 1.16 to 1.80), with substantial heterogeneity in all sub-groups, between 77.3% and 95.3% (Figure 7). In meta-regression analysis, there was a significant difference between albumin cobalt -binding spectrophotometric assays and enzyme-linked immunosorbent assays (*p* = 0.012), and between automatic analysers and enzyme-linked immunosorbent assays (*p* = 0.006), but not between albumin cobalt-binding spectrophotometric assays and automatic analysers (*p* = 0.32) 

#### 3.2.6. Certainty of Evidence

The initial level of certainty for IMA SMD values was considered low because of the cross-sectional nature of the studies (rating 2, ⊕⊕⊝⊝). After considering the low risk of bias in all studies (no rating change required), the extreme and unexplained heterogeneity (downgrade one level), the lack of indirectness (no rating change required), the relatively low imprecision (relatively narrow confidence intervals without threshold crossing, no rating change required), the large effect size (SMD = 2.52, upgrade one level), and the presence of publication bias (downgrade one level), the overall level of certainty was downgraded to very low (rating 1, ⊕⊝⊝⊝).

### 3.3. Intracerebral Haemorrhage

#### 3.3.1. Studies Selected

Six studies reported IMA concentrations in 132 ICH patients and 291 controls [16,27,28,30,34,35].

#### 3.3.2. Risk of Bias

The risk of bias was low in all studies (Table 2).

#### 3.3.3. Results of Individual Studies and Syntheses

**Figure 8:**
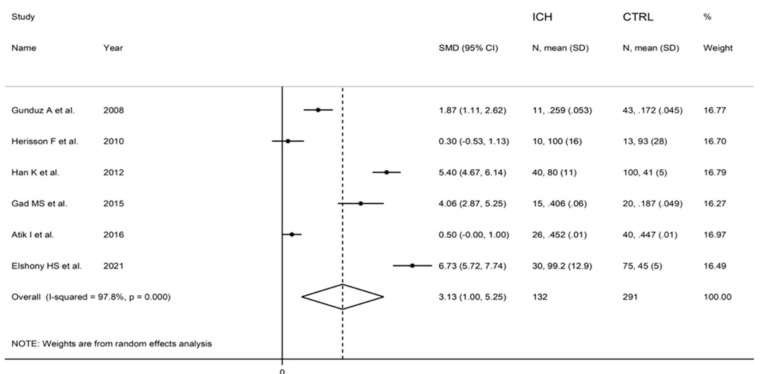
Forest plot of studies examining ischaemia-modified albumin in patients with intracerebral haemorrhage and controls.

**Figure 9:**
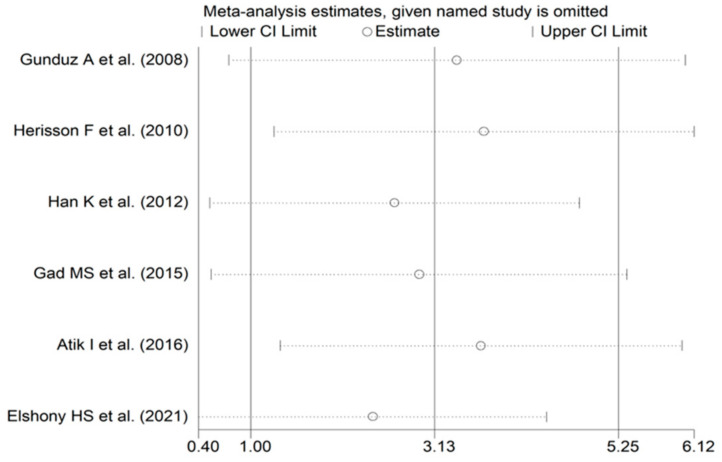
Sensitivity analysis of the association between serum ischaemia-modified albumin and intracerebral haemorrhage. For each study, the displayed effect size (hollow circles) corresponds to an overall effect calculated from a meta-analysis excluding that study.

**Figure 10:**
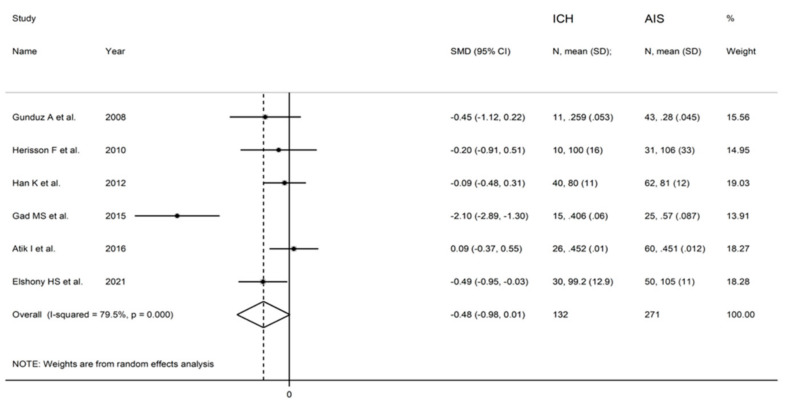
Forest plot of studies examining ischaemia-modified albumin in patients with intracerebral haemorrhage and acute ischaemic stroke.

The forest plot of IMA concentrations in ICH patients and controls is shown in Figure 8. In all studies, ICH patients had higher IMA concentrations (mean difference range, 0.30 to 6.73) although the difference was not significant in two [28,35]. A random-effects model was used given the substantial heterogeneity observed (I^2^ = 97.8%, *p* < 0.001). Pooled results showed that IMA concentrations were significantly higher in ICH patients than controls (SMD = 3.13, 95% CI 1.00 to 5.25; *p* = 0.004). In sensitivity analysis, the corresponding pooled SMD values were not substantially altered when individual studies were sequentially omitted (effect size range, between 2.41 and 3.69; Figure 9). There was a non-significant trend toward lower IMA concentrations in ICH versus AIS patients (SMD = −0.48, 95% CI −0.98 to 0.01; *p* = 0.056; I^2^ = 79.5%, *p* < 0.001) (Figure 10).

#### 3.3.4. Publication Bias

The assessment of publication bias could not be performed because of the small number of studies.

#### 3.3.5. Meta-Regression and Sub-Group Analysis

Meta-regression and sub-group analyses could not be performed because of the small number of studies.

#### 3.3.6. Certainty of Evidence

The initial level of certainty for IMA SMD values was considered low because of the cross-sectional nature of the studies (rating 2, ⊕⊕⊝⊝). After considering the low risk of bias in all studies (no rating change required), the extreme and unexplained heterogeneity (downgrade one level), the lack of indirectness (no rating change required), the relatively low imprecision (relatively narrow confidence intervals without threshold crossing, no rating change required), the large effect size (SMD = 3.13, upgrade one level), and the lack of assessment of publication bias (downgrade one level), the overall level of certainty was downgraded to very low (rating 1, ⊕⊝⊝⊝).

### 3.4. Subarachonid Haemorrhage

#### 3.4.1. Studies Selected

Three studies reported IMA concentrations in 90 SAH patients and 218 controls [16,27,30].

#### 3.4.2. Risk of Bias

The risk of bias was low in all studies (Table 2).

#### 3.4.3. Results of Individual Studies and Syntheses

**Figure 11:**
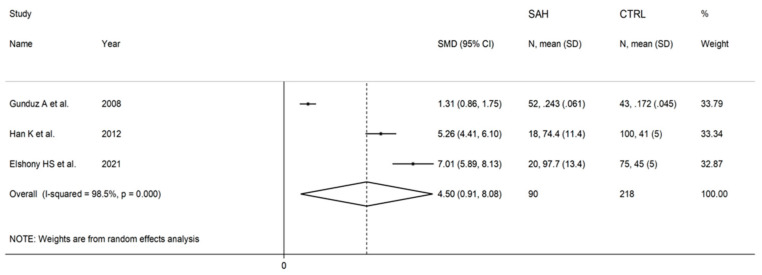
Forest plot of studies examining ischaemia-modified albumin in patients with subarachnoid haemorrhage and controls.

**Figure 12:**
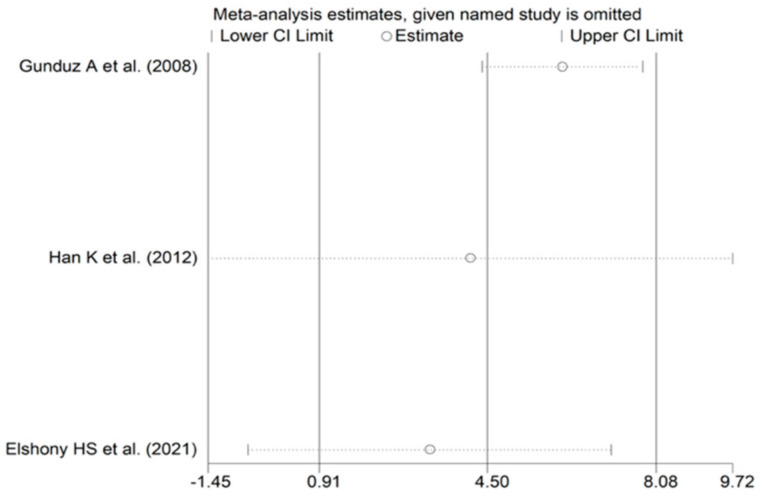
Sensitivity analysis of the association between serum ischaemia-modified albumin and subarachonid haemorrhage. For each study, the displayed effect size (hollow circles) corresponds to an overall effect calculated from a meta-analysis excluding that study.

**Figure 13:**
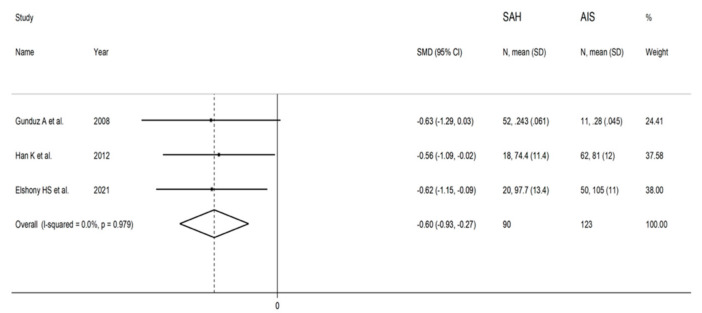
Forest plot of studies examining ischaemia-modified albumin in patients with subarachnoid haemorrhage and acute ischaemic stroke.

**Figure 14:**
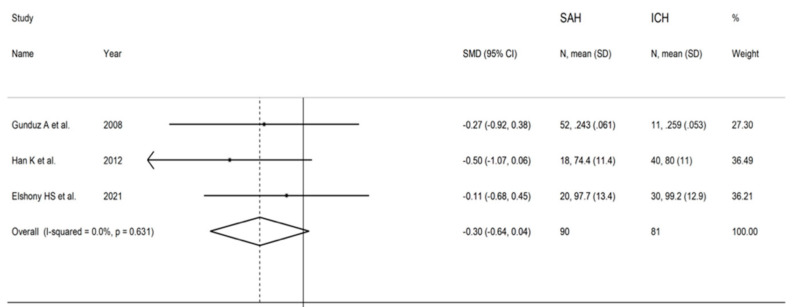
Forest plot of studies examining ischaemia-modified albumin values in patients with subarachnoid haemorrhage and intracerebral haemorrhage.

The forest plot of IMA concentrations in SAH patients and controls is shown in Figure 11. In all studies, SAH patients had significantly higher IMA concentrations (mean difference range, 1.31 to 7.01). Substantial heterogeneity between studies was observed (I^2^ = 98.5%, *p* < 0.001), which justified the use of a random-effects model. Pooled results showed that IMA concentrations were significantly higher in SAH patients than controls (SMD = 4.50, 95% CI 0.91 to 7.01; *p* = 0.014). In sensitivity analysis, the direction of pooled SMD values was not modified when each study was in turn removed (effect size range, between 3.3 and 6.1; Figure 12). IMA concentrations in SAH patients were significantly lower than AIS patients (SMD = −0.60, 95% CI −0.93 to −0.27; *p* < 0.001; I^2^ = 0.0%, *p* = 0.979; Figure 13) but not significantly lower than ICH patients (SMD = −0.30, 95% CI −0.64 to 0.04; *p* = 0.086; I^2^ = 0.0%, *p* = 0.631; Figure 14).

#### 3.4.4. Publication Bias

The assessment of publication bias was not possible because of the small number of studies.

#### 3.4.5. Meta-Regression and Subgroup Analysis

Meta-regression and subgroup analyses could not be conducted because of the small number of studies.

#### 3.4.6. Certainty of Evidence

The initial level of certainty for IMA SMD values was considered low because of the cross-sectional nature of the studies (rating 2, ⊕⊕⊝⊝). After considering the low risk of bias in all studies (no rating change required), the extreme and unexplained heterogeneity (downgrade one level), the lack of indirectness (no rating change required), the relatively low imprecision (relatively narrow confidence intervals without threshold crossing, no rating change required), the large effect size (SMD = 4.50, upgrade one level), and the lack of assessment of publication bias (downgrade one level), the overall level of certainty was downgraded to very low (rating 1, ⊕⊝⊝⊝).

## 4. Discussion

In our systematic review and meta-analysis, serum IMA concentrations within 24 h of symptom onset were significantly higher in patients with AIS, ICH, and SAH, diagnosed according to current guidelines, when compared to non-stroke controls. In studies investigating AIS, meta-regression analysis showed a significant association between effect size and proportion of males but not with other patient or study characteristics. Furthermore, in sub-group analysis, the effect size was relatively larger in studies conducted in Egypt and India when compared to China or Turkey. The relatively small number of studies investigating ICH and SAH did not allow conducting meta-regression or subgroup analyses. Notably, in studies performing direct comparisons between different stroke subtypes, serum IMA concentrations were progressively higher in SAH, ICH, and AIS. Therefore, the results of our study suggest that IMA could be a useful biomarker for the early diagnosis of stroke and to differentiate between SAH, ICH, and AIS, particularly when rapid access to imaging facilities is delayed or not possible.

Albumin undergoes modifications during ischaemic conditions that are associated with a pro-oxidative state, the increased generation of reactive oxygen species, and an acidic environment [42,43,44]. Although the exact chemical reactions involved in such modifications are not fully established, the resulting variant, IMA, has been shown to be transient as it generally reverts to albumin after an ischaemic event. For example, a significant increase in serum IMA concentrations occurred within 10 min of balloon occlusion during a percutaneous coronary intervention and persisted for up to 12 h before returning to baseline after a further 12 h [45]. The magnitude of such changes seems to depend on the duration of the ischaemic process being more prominent after prolonged ischaemia [13]. 

Several colorimetric and immunochemical methods have been developed to measure circulating IMA concentrations. Whilst some of them are relatively simple and have high sensitivity and specificity, particularly the albumin copper-binding assay, the enzyme-linked immunosorbent assay, and the surface plasmon resonance immunosensor, their use is currently limited to small-scale research studies [13]. In our systematic review and meta-analysis, the majority of studies measured IMA using the albumin cobalt-binding method based on the measurement of the binding of cobalt to albumin in serum [46]. Known concentrations of cobalt are added to a serum sample that binds to normal albumin but not to IMA. The unbound cobalt ions react with dithiothreitol, a colourising reagent, to form coloured complexes that can be quantified spectrophotometrically. Whilst extensively used for measuring IMA, this method is not exempt from limitations as conformational changes in albumin due to fluctuations in pH or the presence of denaturing agents, chemicals, or medications, can lead to inaccurate results [13]. Another issue is with regard to the lack of standardization. Most authors express the results as absorbance units, which might depend on investigator experience and/or sensitivity of the equipment [13]. Furthermore, some investigators have used IMA internal standards obtained in their laboratories [13]. Such limitations might account, at least partly, for the between-study variance observed in our meta-analysis, although lack of consensus regarding the exact mechanisms involved in the generation of IMA should also be emphasised in this context. To address these issues, methods for assessing IMA concentrations in biological fluids based on immunological reactions using antibodies to modified albumin have been proposed, although their use remains relatively limited. In our analyses, the use of enzyme-linked immunosorbent assays was associated with a significantly larger effect size when compared to albumin copper-binding spectrophotometric assays or automatic analysers. However, in the selected studies, no information was provided regarding the type of assay used in automatic analysers, which might be theoretically based on modified protocols of the albumin copper-binding assay. This proposition would explain the absence of significant differences in effect size between studies using albumin copper-binding assays and those using automatic analysers.

The role of IMA as a diagnostic biomarker has primarily been investigated in clinically overt ischaemic states, e.g., acute coronary syndrome and AIS. However, a significant increase in serum IMA concentrations has also been reported in other conditions such as heart failure [47], neurodegenerative disorders [48], diabetes [49], pregnancy disorders [50,51,52], and cancer [53]. The results of these studies, together with the evidence of increased IMA concentrations in ICH and SAH [16,27,28,30,34,35], suggest that the likely common denominator for the acute increase in IMA accompanying a wide range of conditions is a state of oxidative stress rather than ischaemia *per se* [54,55]. In the context of stroke, for example, increasing evidence suggests a critical role of oxidative stress in the pathophysiology and clinical progress of ICH and SAH, which might also account for the increased generation of IMA [56,57,58,59].

A previous systematic review and meta-analysis investigated the diagnostic accuracy of IMA in six studies of AIS, reporting a sensitivity of 0.80 (95% CI 0.69 to 0.88), a specificity of 0.80 (95% CI 0.71 to 0.87), and an area under the receiver operating characteristic curve of 0.86 (95% CI 0.83 to 0.89). The control group was represented by subjects without stroke in four studies and various combination of non-stroke, ICH, and SAH participants in the remaining two [60]. Unlike this study, we separately meta-analysed the SMDs of IMA concentrations in patients with AIS, ICH, and SAH vs. non-stroke controls and investigated possible differences in IMA concentrations between stroke subtypes. The mechanisms underlying the reported association, in meta-regression analysis, between effect size and proportion of males require further investigation as a recent study failed to report significant differences in serum IMA concentrations between males ≥45 years (representative of the patients in the studies selected, Table 1) and fertile and post-menopausal females [61]. However, it is also important to emphasise that several studies have reported the presence of gender-associated differences in oxidative stress, with females exhibiting a reduced susceptibility to oxidative stress [62,63]. Furthermore, the relatively larger effect size, in sub-group analysis, in studies conducted in Egypt and India when compared to China or Turkey, suggests possible differences in IMA generation between specific ethnic groups. This proposition is supported by the results of studies reporting significant differences in IMA concentrations between Africans and Caucasians [64,65]. Alike gender, further research is warranted to determine whether possible ethnic-related differences in IMA changes during stroke reflect underlying differences in oxidative stress responses to brain insults [66,67]. Finally, the observation of the relatively higher IMA concentrations in AIS when compared to ICH and SAH, whilst suggesting the potential utility of IMA in discriminating between specific stroke subtypes, require further confirmatory studies given the significant differences in the management of patients with ischaemic vs. haemorrhagic stroke. 

The strengths of our study include the conduct of separate meta-analyses for AIS, ICH, and SAH, the investigation, when possible, of associations between the effect size and several patient and study characteristics using meta-regression and subgroup analysis, and the assessment of the certainty of evidence using GRADE. One important limitation is represented by the substantial between-study heterogeneity. However, it is also important to emphasise that in sensitivity analysis, the effect size was not substantially affected when individual studies were in turn removed.

## 5. Conclusions

Our systematic review and meta-analysis have shown the presence of significant differences in serum IMA concentrations between patients with specific stroke subtypes and non-stroke controls. Additional research is warranted to investigate the relationships between IMA generation and the extent of brain damage, clinical progress, long-term outcomes, and specific patient characteristics such as gender and ethnicity. Only then can the clinical utility of routine IMA measurements be appropriately determined.

## Figures and Tables

**Table 1:** Study characteristics.

	Controls				Patients with Stroke					
								AIS	ICH	SAH
First Author and Year, Country [Ref]	N	Age * (Years)	M/F	IMA Mean ± SD	N	Age * (Years)	M/F	IMA Mean ± SD	IMA Mean ± SD	IMA Mean ± SD
Gunduz A et al. 2008, Turkey [27]	43	57	NR	0.172 ± 0.045 (ABSU)	43	71	23/20	0.280 ± 0.045 (ABSU)	0.259 ± 0.053 (ABSU)	0.243 ± 0.061 (ABSU)
Herisson F et al. 2010, France [28]	13	49	5/8	93 ± 28 (U/mL)	31	59	20/11	106 ± 33 (U/mL)	100 ± 16 (U/mL)	-
Ahn JH et al. 2011, South Korea [29]	24	NR	NR	86.5 ± 8.5 (U/mL)	28	NR	NR	107.4 ± 11.0 (U/mL)	-	-
Han K et al. 2012, China [30]	100	61	53/47	41 ± 5 (U/mL)	62	59	38/24	81 ± 12 (U/mL)	80 ± 11 (U/mL)	74.4 ± 11.4 (U/mL)
Ertekin B et al. 2013, Turkey [31]	30	52	14/16	0.82 ± 0.13 (ABSU)	30	66	12/18	1.18 ± 0.22 (ABSU)	-	-
Çakmak VA et al. 2014, Turkey [32]	30	65	20/10	0.305 ± 0.08 (ABSU)	38	66	20/18	0.539 ± 0.18 (ABSU)	-	-
Can S et al. 2015, Turkey [33]	34	58	18/16	0.35 ± 0.04 (ABSU)	50	68	26/24	0.52 ± 0.25 (ABSU)	-	-
Gad MS et al. 2015, Egypt [34]	20	NR	NR	0.187 ± 0.049 (ABSU)	25	NR	NR	0.57 ± 0.087 (ABSU)	0.406 ± 0.060 (ABSU)	-
Atik I et al. 2016, Turkey [35]	40	NR	NR	0.447 ± 0.010 (ABSU)	60	NR	30/30	0.451 ± 0.012 (ABSU)	0.452 ± 0.010 (ABSU)	-
Kumar A et al. 2016, India [36]	56	NR	NR	324 ± 36 (ABSU)	56	NR	46/10	489 ± 25 (ABSU)	-	-
Gudi JG et al. 2017, India [37]	30	NR	NR	0.16 ± 0.03 (ABSU)	60	NR	NR	0.22 ± 0.03 (ABSU)	-	-
Jena I et al. 2017, India [38]	50	55	28/22	70.7 ± 8.4 (U/mL)	50	60	26/24	97.6 ± 13.6 (U/mL)	-	-
Menon B et al. 2018, India [15]	50	56	30/20	79 ± 6.3 (IU/mL)	50	58	39/11	108 ± 8.9 (IU/mL)	-	-
Yilmaz AB et al. 2017, Turkey [39]	85	70	44/41	66.8 ± 9.6 (g/dL)	143	72	76/67	79.4 ± 11.9 (g/dL)	-	-
Ma J et al. (a) 2018, China [40]	114	54	54/60	1.2 ± 0.5 (pg/mL)	92	54	48/44	1.9 ± 0.6 (pg/mL)	-	-
Ma J et al. (b) 2018, China [40]	114	54	54/60	1.2 ± 0.5 (pg/mL)	108	54	55/53	2.0 ± 0.4 (pg/mL)	-	-
Elshony HS et al. 2021, Egypt [16]	75	NR	NR	45 ± 5 (U/mL)	50	NR	NR	105 ± 11 (U/mL)	99.2 ± 12.9 (U/mL)	97.7 ± 13.7 (U/mL)
Zhong C et al. 2021, China [41]	80	62	48/32	75 ± 4 (U/mL)	160	62	86/74	82 ± 4 (U/mL)	-	-

Legend: NR, not reported; ABSU, absorbance units; IU, international units; U, units; *, mean or median.

**Table 2:** The Joanna Briggs Institute critical appraisal checklist.

Study	Were the Criteria for Inclusion Clearly Defined?	Were the Subjects and the Setting Described in Detail?	Was the Exposure Measured in a Valid and Reliable Way?	Were Objective, Standard Criteria Used for Measurement of the Condition?	Were Confounding Factors Identified?	Were Strategies to Deal with Confounding Factors Stated?	Were the Outcomes Measured in a Valid and Reliable Way?	Was Appropriate Statistical Analysis Used?	Risk of Bias
Gunduz A [27]	Yes	Yes	Yes	Yes	No	No	Yes	No	Low
Herisson F [28]	Yes	Yes	Yes	Yes	No	No	Yes	No	Low
Ahn JH [29]	Yes	Yes	Yes	Yes	No	No	Yes	No	Low
Han K [30]	Yes	Yes	Yes	Yes	No	No	Yes	No	Low
Ertekin B [31]	Yes	Yes	Yes	Yes	No	No	Yes	No	Low
Çakmak VA [32]	Yes	Yes	Yes	Yes	No	No	Yes	No	Low
Can S [33]	Yes	Yes	Yes	Yes	No	No	Yes	No	Low
Gad MS [34]	Yes	Yes	Yes	Yes	No	No	Yes	No	Low
Atik I [35]	Yes	Yes	Yes	Yes	No	No	Yes	No	Low
Kumar A [36]	Yes	Yes	Yes	Yes	No	No	Yes	No	Low
Gudi JG [37]	Yes	Yes	Yes	Yes	No	No	Yes	No	Low
Jena I [38]	Yes	Yes	Yes	Yes	No	No	Yes	No	Low
Menon B [15]	Yes	Yes	Yes	Yes	No	No	Yes	No	Low
Yilmaz AB [39]	Yes	Yes	Yes	Yes	No	No	Yes	No	Low
Ma J [40]	Yes	Yes	Yes	Yes	Yes	Yes	Yes	Yes	Low
Elshony HS [16]	Yes	Yes	Yes	Yes	No	No	Yes	No	Low
Zhong C [41]	Yes	Yes	Yes	Yes	No	No	Yes	No	Low

## Data Availability

The data that support the findings of this systematic review and meta-analysis are available from the corresponding author, A.Z., upon reasonable request.

## Supplementary Materials

The following are available online at https://www.mdpi.com/article/10.3390/biom12050653/s1, Table S1: PRISMA 2020 abstract checklist; Table S2: PRISMA 2020 manuscript checklist.
